# Widespread Recurrence 7 Years after Radical Abdominal Trachelectomy for Early Cervical Adenocarcinoma

**DOI:** 10.1155/2015/517496

**Published:** 2015-03-30

**Authors:** M. Coskun Salman, Nejat Ozgul, Kunter Yuce

**Affiliations:** Department of Obstetrics and Gynecology, Hacettepe University Faculty of Medicine, 06100 Ankara, Turkey

## Abstract

Cervical cancer is the third most common female cancer worldwide and the use of routine screening resulted in earlier stage and younger age at diagnosis. Fertility preservation via radical trachelectomy comes up as an option in such patients. Recent literature reviews confirm the safety of this operation with excellent oncologic outcomes in appropriately chosen patients. However, recurrent disease is likely and a strict follow-up is recommended to detect recurrences at an early stage following radical trachelectomy. In this report, a case who underwent radical trachelectomy and developed widespread recurrences 7 years after initial surgery possibly due to the lack of oncologic follow-up is discussed.

## 1. Introduction

Cervical cancer is the third most common female cancer worldwide with more than half a million new cases diagnosed and 274,000 deaths occurred annually [1]. Nevertheless, the use of cervical cancer screening via Pap test as recommended resulted in earlier stage and younger age at diagnosis especially in developed countries. In USA, among patients diagnosed with invasive cervical carcinoma, 28% are less than 40 years of age. Furthermore, 39% of patients with stage 1 disease are younger than 40 [2]. The traditional treatment for early cervical cancer is radical hysterectomy and up to 90–95% 5-year survival is expected following such treatment in patients with stage 1A2-1B1 disease [3]. However, this approach is associated with permanent loss of fertility that may cause significant morbidity in form of depression, anxiety, grief, and sexual dysfunction [4]. Accordingly, fertility preservation via radical trachelectomy comes up as an option in these patients. It was shown that almost 50% of the patients with cervical cancer who are less than 40 years of age are eligible for radical trachelectomy and this approach has become increasingly accepted over the past 20 years [2, 5]. Recent literature reviews confirm the safety of this operation with excellent oncologic outcomes and some authors now consider radical trachelectomy as a standard of care for appropriately chosen patients [6, 7]. On the other hand, a careful and strict follow-up programme is recommended to detect recurrences at an early stage after radical trachelectomy [8].

Here, a case who developed widespread recurrence 7 years after radical abdominal trachelectomy possibly due to the lack of oncologic follow-up is reported. Fertility-sparing surgery in cervical cancer was discussed as well with special emphasis on eligibility criteria, indications for completion surgery and/or adjuvant radiotherapy, oncological safety, pregnancy outcomes, and postoperative follow-up recommendations.

## 2. Case Report

A 35-year-old nulliparous woman was referred to our hospital from an in vitro fertilization center for pelvic mass and adenocarcinoma on Pap test. The review of her past medical history revealed that she had been diagnosed to have stage 1B1 cervical adenocarcinoma 6 years ago when she had presented with complaints of postcoital bleeding and bloody vaginal discharge. On her examination, she had been found to have an exophytic lesion involving the central part of the cervix and protruding into the vagina. The outer parts of the exocervix and vaginal fornices had looked normal. A punch biopsy of the lesion had shown invasive adenocarcinoma and the patient had been subjected to a radical abdominal trachelectomy with pelvic and para-aortic lymphadenectomy without any further diagnostic evaluation. On her permanent pathology, the disease was a grade 2 adenocarcinoma which was 1.5 cm in diameter with no vaginal or parametrial involvement. Excised vagina was 2.5 cm in length while the excised parametria was 3.0 and 2.8 cm in width at the left and right sides, respectively. Also, 29 lymph nodes were negative for metastasis and all surgical margins were free of disease. However, there was diffuse lymphovascular space invasion and the disease invaded the cervical stroma deeply with 75% of stromal invasion detected in some areas. The patient and her husband had rejected adjuvant pelvic radiation treatment which had been recommended due to those two intermediate pathological risk factors. Despite strict follow-up recommendations, she had been lost to follow-up thereafter. During the subsequent period, she failed spontaneous conception for the first postoperative year. Then, 6 cycles of controlled ovarian hyperstimulation with intrauterine insemination (COH + IUI) were done but failed. Lastly, four in vitro fertilization cycles were performed without any success. Her conception attempts lasted for 5 years in total and during that period the patient was not subjected to any investigations for her cervical cancer and she did not meet with any gynecologic oncologist.

**Figure 1:**
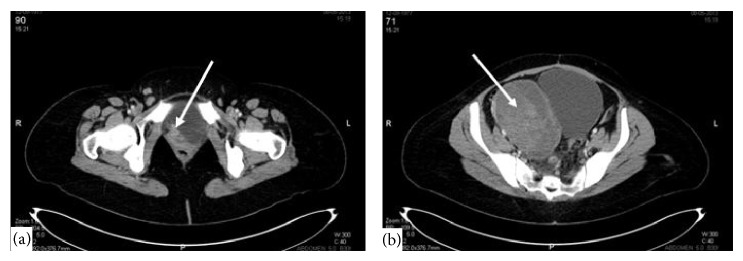
Cervical mass invading bladder (a) and right adnexal heterogeneous mass (b) on computerized tomography.

**Figure 2:**
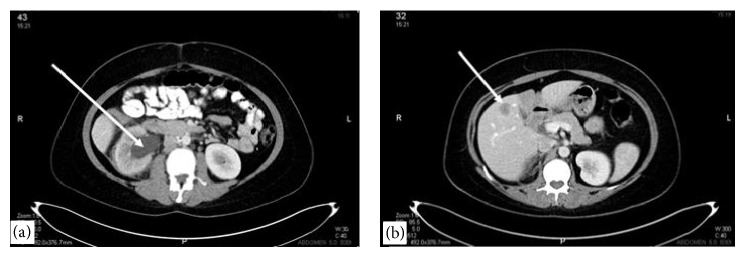
Computerized tomography showing severe hydroureteronephrosis on right side (a) and one of the hepatic metastases (b).

On her admission, the patient had abnormal vaginal bleeding lasting for more than 1 year with severe pelvic pain, gross hematuria, right lower extremity pain, and swelling. On pelvic examination, the residual cervix was completely infiltrated with tumor. In addition, the upper anterior vagina and lateral fornices were involved. The right parametrium was infiltrated with tumor extending to the pelvic sidewall and a right adnexal mass was palpated. Under general anesthesia, a cervical biopsy was done which revealed recurrent adenocarcinoma. Cystoscopy was done for gross hematuria during the same operative session which showed neoplastic invasion causing a mass within the bladder. Cystoscopic biopsy from the bladder mass confirmed adenocarcinoma infiltration. After assuring that cervical adenocarcinoma has recurred, imaging studies were performed to document the extent of recurrence. Computerized tomography of abdomen and thorax and magnetic resonance imaging of pelvis showed recurrent cervical mass invading upper vagina and bladder with right parametrial infiltration up to pelvic sidewall (Figure 1(a)). A right adnexal heterogeneous mass measuring 11 × 9 cm was seen (Figure 1(b)). Severe hydronephrosis on the right side (Figure 2(a)) and multiple hepatic metastases (Figure 2(b)) were detected. Thorax was free of metastatic involvement. Due to multiple distant metastases and unresectable locoregional recurrence, surgery was not considered. Combination chemotherapy consisting of paclitaxel and carboplatin was initiated following the placement of right percutaneous nephrostomy. After 6th cycle of chemotherapy, the patient had significant symptomatic improvement. Her last visit was five months after the completion of chemotherapy when she was alive with disease.

## 3. Discussion

More than 40% of all patients diagnosed with invasive cervical cancer are of childbearing age and a significant proportion of these young women are candidates for fertility-sparing surgery [2, 9]. For such patients, Daniel Dargent described the first successful fertility-sparing surgery in 1986 which was radical vaginal trachelectomy combined with laparoscopic lymphadenectomy [10]. Abdominal technique for radical trachelectomy was first published by Smith et al. almost 10 years later [11]. To date, the safety and feasibility of fertility-sparing surgery in cervical cancer were supported by several case series in terms of both oncological and fertility outcomes. This applies to both vaginal and abdominal approaches [4]. In total, more than 600 cases of radical trachelectomies may be determined when published literature is reviewed. In this patient population, the overall recurrence rate is less than 5% and mortality rate is less than 3%. These recurrence and mortality rates are comparable to those of patients who were subjected to traditional radical hysterectomy [6]. Nevertheless, the risk of recurrence is higher if the tumor size exceeds 2 cm in diameter or if lymphovascular space invasion is present [4, 6]. Thereby, the indications for radical trachelectomy include strong fertility desire with no evidence of infertility in women aged less than 40 years, disease stage 1A1 with lymphovascular space invasion, 1A2 or 1B1 when tumor size is less than 2 cm, limited endocervical involvement, and no evidence of pelvic lymph node metastasis or distant metastasis. Trachelectomy is acceptable for both glandular and squamous lesions, but unfavorable histology such as neuroendocrine carcinoma should be excluded [12]. Our patient was 29 years old during the initial diagnosis and had a stage 1B1 disease without any evidence of extracervical disease. Also, her tumor was 1.5 cm in diameter. She was recently married and nulligravid and had strong desire to maintain her future childbearing potential without any signs or symptoms of impaired fertility. Thus, she was considered as a good candidate for radical trachelectomy.

On the other hand, although all the above-mentioned eligibility criteria are met, the pathological assessment of the radical trachelectomy specimen will identify that fertility preservation is not appropriate in 30% of patients to be able to ensure oncologic outcomes [7]. During the preoperative process, the patient and her family should be informed about the risks of immediate radical hysterectomy and/or need for postoperative adjuvant radiation therapy which are not compatible with the preservation of fertility [13]. The pathological evaluation of surgical specimen in our patient revealed deep cervical stromal invasion and diffuse lymphovascular space invasion which are considered to be pathological intermediate risk factors [14]. In patients with 1 or 2 intermediate risk factors, adjuvant external pelvic radiation with or without cisplatin-based chemotherapy is recommended since such an approach confers significant advantage in terms of both disease-free and overall survival [14–16]. This is a common practice in adenocarcinoma as well in spite of the fact that it is relatively resistant to radiation compared to squamous histology. Therefore, adjuvant radiation therapy was recommended, but our patient and her husband refused any further treatment due to concerns about its conflict with the preserved fertility.

Following radical trachelectomy, 40% of recurrences occur in the parametrium or pelvic sidewall whereas 25% occur in the pelvic, para-aortic, and/or supraclavicular lymph nodes [6]. Hence, the patients should comply with a strict follow-up schedule to be able to detect recurrences at an early stage. Assessment with magnetic resonance imaging, cytology, and colposcopy should be carried out at the end of 6 months and contraception is recommended until then. Attempts to conceive are allowed if there is no evidence of recurrence. Follow-up is continued once every 3 months for 1 year, once every 4 months for the second year, and every 6 months thereafter. Yearly follow-up should be maintained up to 10 years [8]. Despite follow-up recommendations, our patient was lost to follow-up after discharge from hospital. She had failed spontaneous and assisted conception attempts for years without any investigations for cervical cancer.

As a result, she was diagnosed to have local, regional, and distant recurrences 7 years after initial diagnosis. Due to multiple sites of unresectable recurrent disease surgery was not an option and combination chemotherapy was initiated which resulted in considerable symptomatic improvement.

In conclusion, fertility preservation may be considered in well-selected young women with invasive cervical carcinoma. However, a careful and detailed discussion before surgery with the patient and her family is of vital importance. This discussion should include the possibilities of completion surgery and adjuvant radiation therapy which conflict with future fertility. Also, the family should be informed about the risk and pattern of recurrent disease and the gynecologist should make sure that the patient will comply with the predetermined strict follow-up schedule. Otherwise, attempts to preserve fertility may lead to catastrophic, life-threatening consequences as seen in the current case.
